# Supplementary material for: Aronia Berry Supplementation Mitigates Inflammation in T Cell Transfer-Induced Colitis by Decreasing Oxidative Stress
Source: Nutrients. 2019 Jun 12;11(6):1316. doi: 10.3390/nu11061316 (PMC6627224; doi:10.3390/nu11061316)
Supplement: Supplementary file 1 [file nutrients-11-01316-s001.pdf]

Supplementary Material

Methods

mRNA analysis

RNA in colon, spleen and MLN was extracted by TRIzol reagent following the manufacturer's recommended protocol. Colon samples were mixed with TRIzol reagent and homogenized with a Fisher Scientific™ Bead Mill 4 homogenizer (Pittsburgh, PA). MLN and spleen samples were homogenized by pipetting up and down in TRIzol reagent. After that, chloroform was added for phase separation, and aqueous phase was removed and mixed with isopropanol to precipitate RNA. RNA was pelleted by centrifugation, washed with 75% ethanol, and dissolved in RNase-free water. The extracted RNA was further purified using an RNeasy mini kit (Qiagen, Valencia, CA). Briefly, the RNA solution was mixed with the supplied lysis buffer and ethanol, and then loaded onto a column, digested with an RNase-free DNase and RNA was eluted with RNase-free water. RNA concentration was then determined by fluorometric quantitation on the Qubit (Life Technologies, Carlsbad, CA). Then, cDNA was synthesized from 1 µg RNA using an iScript™ cDNA synthesis kit (Bio-Rad, Hercules, CA) on a Veriti® thermal cycler (Life Technologies, Carlsbad, CA), resulting in a 20 µL reaction volume. Then, 5 µL of the newly synthesized cDNA product was pooled for preparation of a standard curve.

Reverse transcription quantitative PCR (RT-qPCR) was performed using the iTaq™ Universal SYBR® Green Supermix (Bio-Rad, Hercules, CA), on a Bio-Rad CFX96 system (Bio-Rad, Hercules, CA), following the manufacture's protocol. Specific primers were designed by the NCBI primer design tool to span an intron-exon boundary of genes [1]. The genes and primer sequences determined by RT-qPCR are listed in **Supplementary Table S1**. The controls consisted of *Eef2* and *Rplp0* genes that are stably expressed in colitic mice [2,3]. The RT-qPCR reaction volume was 10 µL, consisting of 4 µL of 20-times diluted cDNA and 6 µL of master mix (5 µL of SYBR® Green Supermix; 0.5 µL of each 2.5 µM primer). Each RT-qPCR run included no cDNA and no reverse transcriptase as negative controls. A 6-point standard curve was prepared using the 4-fold serial dilution of the samples cDNA pool and the unknown samples were quantified by the relative standard curve method using Bio-Rad CFX manager 2.1 software (Bio-Rad, Hercules, CA) [4].

Supplementary Table S1 Primer sequences of target and reference genes.

Name	GenBank accession number	Forward (primer sequence 5′–3′)	Reverse (primer sequence 5′–3′)
Eef2	NM_007907.2	CCC CAA CAT TCT CAC CGA CA	AGA GCG CCC TCC TTA GTA GC
Rplp0	NM_007475.5	CTC GTT GGA GTG ACA TCG TCT	GAT CTG CTG CAT CTG CTT GG
Foxp3	NM_054039.2	GAT CCC CCT CTA GCA GTC CA	TCA GAG GCA GGC TGG ATA AC
Rorc	NM_011281.3	AAG GAC GGC ACC AAG GGA G	CAA TTT GTG AGG TGT GGG TCT TC
Il17a	NM_010552.3	TCA TCT GTG TCT CTG ATG CTG TT	TTG GCC TCA GTG TTT GGA CA
Il22	NM_016971.2	GTT GAC ACT TGT GCG ATC TCT G	TAG CAC TGA TCC TTA GCA CTG AC
Il6	NM_031168.2	TCC AGT TGC CTT CTT GGG AC	GCC ATT GCA CAA CTC TTT TCT CA
Il10	NM_010548.2	CTT TAA GGG TTA CTT GGG TTG CC	GCT CCA CTG CCT TGC TCT TA
Ifng	NM_008337.4	GCT ACA CAC TGC ATC TTG GC	CAT GTC ACC ATC CTT TTG CCA G
Tnf	NM_013693.3	ATG GCC TCC CTC TCA TCA GT	TGG TTT GCT ACG ACG TGG G
Nfe2l2	NM_010902.4	CCC AGC AGG ACA TGG ATT TGA	AGC TCA TAG TCC TTC TGT CGC
Gclc	NM_010295.2	GCA CAT CTA CCA CGC AGT CA	GTC GGA TGG TTG GGG TTT GT
Gsr	NM_010344	GTG GCA CTT GCG TGA ATG TT	ATA GAT GGT GTT CAG GCG GC
Sod2	NM_013671.3	GAA CAA TCT CAA CGC CAC CG	CCA GCA ACT CTC CTT TGG GTT
Gpx1	NM_008160.6	GAA GGC TCA CCC GCT CTT TA	ACA CCG GAG ACC AAA TGA TGT
Gpx2	NM_030677.2	CCA GTT CGG ACA TCA GGA GAA	GTC ATG AGG GAG AAC GGG TC
Prdx1	NM_011034.4	TGT CAT CTG GCA TGG ATT AAC AC	AAT GGT GCG CTT GGG ATC TG
Slc2a	NM_011400.3	GGC TTG CTT GTA GAG TGA CG	AAC TCC TCA ATA ACC TTC TGG GG
Slc5a1	NM_019810.4	GAT CAT GCT GGT GGG GTC TT	ATC GCT GCA CAA TGA CCT GA

Eef2, encoding eukaryotic translation elongation factor 2; *Rplp0*, encoding ribosomal protein large P0; *Foxp3*, encoding forkhead box P3; *Rorc*, encoding RAR-related orphan receptor gamma; *Il17a*, encoding interleukin 17A; *Il22*, encoding interleukin 22; *Il6*, encoding interleukin 6; *Il10*, encoding interleukin 10; *Ifng*, encoding interferon-γ; *Tnf*, encoding tumor necrosis factors, or TNF; *Nfe2l2*, encoding (erythroid-derived 2)-like 2, or Nrf2; *Gclc*, encoding glutamate-cysteine ligase-catalytic subunit, or GCLC; *Gsr*, encoding glutathione reductase; *Sod2*, encoding superoxide dismutase 2, mitochondrial, or SOD2; *Gpx1*, encoding glutathione peroxidase 1; *Gpx2*, encoding glutathione peroxidase 2; *Prdx1*, encoding peroxiredoxin 1; *Slc2a*, encoding solute carrier family 2 (facilitated glucose transporter), member 1, or GLUT1; *Slc5a1*, encoding solute carrier family 5 (sodium/glucose cotransporter), member 1 (Slc5a1), or SGLT1.

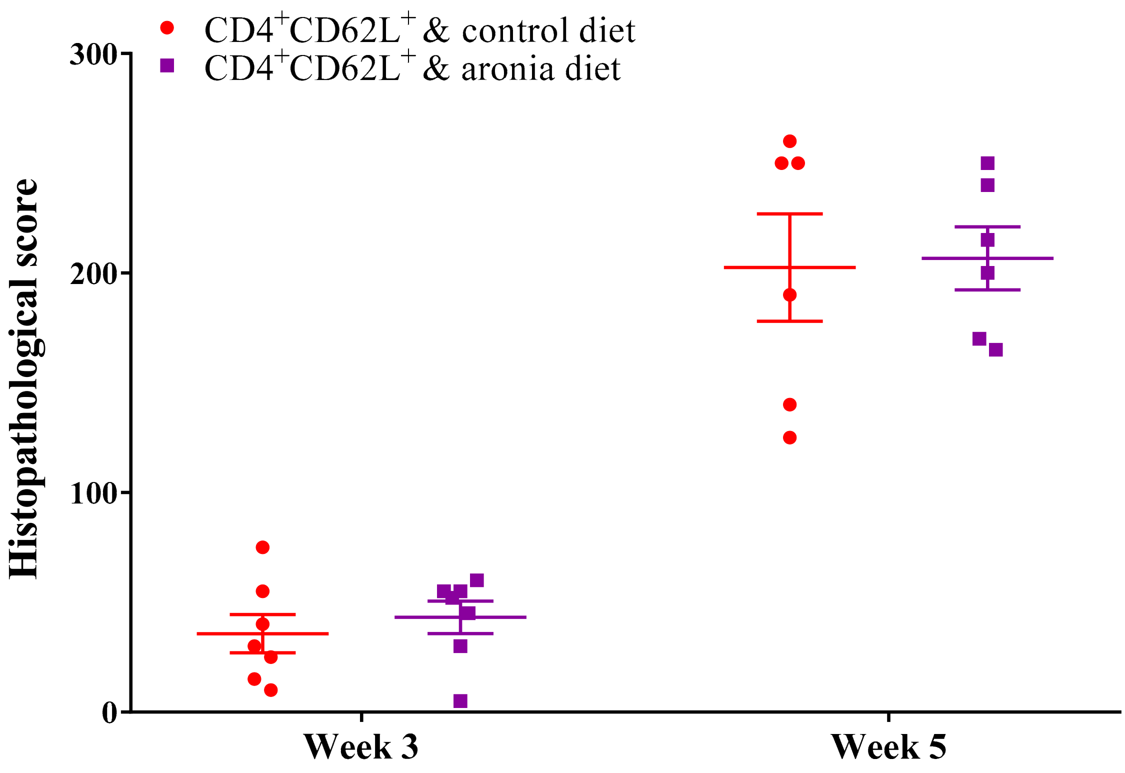

Figure S1. Splenic CD4⁺CD62L⁺ cells from C57BL/6J mice were transferred to Rag1$^{-/-}$ mice**.** Mice consumed the control (AIN-93M) or aronia-supplemented diets for 3 or 5 weeks. Histopathological scores of colons based on degree of inflammatory infiltration of the mucosa, submucosa, muscularis, and serosa, occurrence of crypt exudate, crypt loss, and/or effacement, presence of neutrophils and mutinucleate giant cells in the inflammatory cell infiltrate, and ulceration. Total scores of 0 (unaffected) to 300 (severe, 100% of colon) were possible. (n = 5–7 per group). (Adapted from data reported in our previous publication: [5]).

References:

1. Altschul, S.F.; Gish, W.; Miller, W.; Myers, E.W.; Lipman, D.J. Basic local alignment search tool. *J. Mol. Biol.* **1990**, *215*, 403-410.
2. Eissa, N.; Hussein, H.; Wang, H.X.; Rabbi, M.F.; Bernstein, C.N.; Ghia, J.E. Stability of reference genes for messenger rna quantification by real-time pcr in mouse dextran sodium sulfate experimental colitis. *PLoS One* **2016**, *11*.
3. Eissa, N.; Kermarrec, L.; Hussein, H.; Bernstein, C.N.; Ghia, J.E. Appropriateness of reference genes for normalizing messenger rna in mouse 2,4-dinitrobenzene sulfonic acid (dnbs)-induced colitis using quantitative real time pcr. *Sci. Rep.* **2017**, *7*.
4. Grala, T.M.; Roche, J.R.; Kay, J.K.; Rius, A.G.; White, H.M.; Donkin, S.S.; Littlejohn, M.D.; Snell, R.G.; Phyn, C.V. The expression of genes involved in hepatic metabolism is altered by temporary changes to milking frequency. *J. Dairy Sci.* **2014**, *97*, 838-850.
5. Pei, R.; Martin, D.A.; Valdez, J.; Liu, J.; Kerby, R.; Rey, F.; Smyth, J.; Liu, Z.; Bolling, B.W. Dietary prevention of colitis by aronia berry is mediated through t cell il-10 and increased th17 and treg. *Mol. Nutr. Food Res.* **2019**, *63*, e1800985.
